# A study with cancer stem cells and three-dimensional tumoroids: investigation of the combined effects of 5-fluorouracil and doxorubicin in breast cancer

**DOI:** 10.1007/s12032-024-02423-4

**Published:** 2024-06-23

**Authors:** Seçil Erden Tayhan

**Affiliations:** Faculty of Pharmacy, Department of Pharmaceutical Biotechnology, Tokat Gaziosmanpasa University, Tokat, Turkey

**Keywords:** Breast cancer, Cancer stem cells, Polychemotheraphy, Tumor spheroids, MACS

## Abstract

The purpose of the present study was in vitro determination of the combined effects of doxorubucin and 5-fluorouracil by 2D and 3D culture conditions on breast cancer using MCF-7 cell line and CSCs isolated from these cells. In the first stage of this study, CSC isolation and their characterization were performed. In the next experimental period, the antiproliferative effects of 5-Fu and Dox on the MCF-7 and CSCs were demonstrated on 2D. To evaluate the synergistic/antagonistic effects of these chemotherapeutics, the CI was calculated. Additionally, 3D tumor spheroids were used as another model. In the last step, qRT-PCR analysis was performed to examine apoptosis-related gene expressions. In this study, it was clearly seen that CSCs obtained from the breast cancer cell line express stemness factors. In addition, the antiproliferative effects of 5-Fu and Dox on breast cancer and associated CSCs were very clear. Their synergistic effects were determined by CI values. Moreover, it was seen that combined theraphy changed the expression levels of genes related to apoptosis. Additionally, it was molecularly demonstrated that 3D tumoroids were more resistant than the others. In conclusion, the polychemotherapeutic approach was much more effective than the monotherapy. The fact that this effect was seen not only in breast cancer cells, but also in breast cancer stem cells. In addition, it was very promising that the results obtained were similar in both two-dimensional and three-dimensional tumoroids.

## Introduction

Breast cancer (BC) is the leading cancer cause of death among women all over the world [1]. Chemotherapeutic combinations (polychemotherapy) have recently included in treatment plans of its [2, 3]. Breast stem cells are now associated with cancer stem cells (CSC), which are known to be highly effective in oncological processes and have been identified in many types of cancer [4]. It is known that cancer stem cells are among the main factors that cause drug resistance, metastasis and recurrence [5]. Drug resistance is a major challenge in breast cancer (BC) treatment at present. Accumulating studies indicate that breast cancer stem cells (BCSCs) are responsible for the BC drugs resistance, causing relapse and metastasis in BC patients. Thus, BCSCs elimination could reverse drug resistance and improve drug efficacy to benefit BC patients. As in all types of cancer, many studies are being carried out today to neutralize these powerful cells in breast cancer and to ensure permanent success in cancer treatment [4, 5]. Metastasis, which involves in a wide systematic process, makes treatment difficult and sometimes even impossible [6]. Therefore, the CSC-metastasis relationship has attracted much attention recently. All the aforementioned BCSCs properties may be potential targets to designing a more efficient therapy to use alone or in combination with current used therapies, for the treatment of patients diagnosed with BC.

When preclinical studies conducted to improve treatment effectiveness in BC are examined, in addition to the polychemotherapy and CSC approaches mentioned above, three-dimensional (3D) tumor modeling applications have been frequently encountered recently. The 3D approach makes significant contributions to the transition from the laboratory to the clinic and allows tumor physiology to be examined in a way closest to in vivo [7]. It is known that preclinical tests, which proceed with the results obtained in two-dimensional (2D) monolayer cultures, which are used as the traditional method in in vitro cancer research, cannot guide the clinical phase with high reliability [8]. The vast majority of drugs (90%), which successfully passed preclinical testing, failed in the clinical phases. Therefore, the use of 3D cancer models has both ethical and economic impact in drug screening and development. Therefore, interest in 3D models is increasing in studies on breast cancer. The aim of in vitro cancer models generally is to mimic BC in vivo for a better understanding of tumor physiology, characterization of drug resistance mechanisms and the selection of effective drugs for treatment [9]. In this context, many different 3D models have been developed and one of the most widely used among these models is multicellular tumor spheroids. These spheroids are generally formed from the same type of cells under weak or non-adherent conditions [10].

The inclusion of cancer stem cells in the study allows the results obtained to be used efficiently in different areas of basic and clinical oncology. In addition, to the best of our knowledge, no comparative study on the effects of a strategy in the form of a dual combination of 5-Fu and Dox on Her2-/ER + breast cancer and stem cells has been found in the literature. The fact that the results obtained were supported by molecular analyzes increased the reliability of the study. It is thought that the approaches presented with these mentioned aspects will contribute to the advancement of knowledge in the field of breast cancer treatment.

In this study, the possible effects of the polychemotherapeutic approach shaped by doxorubucin and 5-fluorouracil on breast cancer were investigated in in *vitro* conditions through the MCF-7 breast cancer cell line. In this context, 3D tumor spheroids were created using cancer stem cells isolated from MCF-7, and the effects of combined chemotherapeutics were comprehensively investigated at the cellular and molecular level.

## Materials and methods

### Breast *cancer* stem cell ısolation and characterization

In the first stage of this study, cancer stem cells and their characterization were performed. The reason why the MCF-7 cell line used in this isolation was chosen is that it is negative for human epidermal growth factor receptor 2 (HER2) and positive for estrogen receptor (ER)[10]. Because this type of BC is quite common and can cause considerable mortality [11].

Magnetic-activated cell sorting (MACS) was used to isolate cancer stem cells from the MCF-7 cell line. In this system, CD24 and CD44 antibodies, known as specific markers on breast cancer stem cells (BC-CSCs), were used and CD24^−^ and CD44^+^ cell populations were separated from the others [12, 13]. During isolation, the procedure provided by the company from which the MACS system was commercially supplied (Miltenyi Biotec, Bergisch Gladbach, Germany) was followed exactly.

BC-CSCs were characterized by quantitative real-time polymerase chain reaction (qRT-PCR) by looking at the expression levels of stemness related transcription factors known as SOX2, OCT4, Nanog and Nestin [14]. In addition, since CD24 and CD44 antibodies were used in the MACS separation system used in BC-CSCs isolation, the expression levels in these gene regions were also examined. (Table 1).

**Table 1:** Primer sequences used in BC-CSC characterization

Gene Names	Forward	Reverse
GAPDH	5’-GGATTTGGTCGTATTGGG-3’	5’-GGAAGATGGTGATGGGATT-3’
CD44	5’-CTGCAGGTATGGGTTCATAG-3’	5’-ATATGTGTCATACTGGGAGGTG-3’
CD24	5`-CTCCTACCCACGCAGATTTATTC-3’	5`-AGAGTGAGACCACGAAGAGAC-3’
Nestin	5’-ATCGCTCAGGTCCTGGAA-3’	5’-AAGCTGAGGGAAGTCTTGGA-3’
OCT4	5’-CCTGAAGCAGAAGAGGATCA-3’	5’-CCGCAGCTTACACATGTTCT-3’
SOX2	5’-TACAGCATGTCCTACTCGCAG-3’	5’-GAGGAAGAGGTAACCACAGGG-3’
Nanog	5’-AATAACCTTGGCTGCCGTCT-3’	5’-AGCCTCCCAATCCCAAACAAT-3’

“Comparative ΔΔCT” method was used for the quantification of amplification products obtained by real-time PCR [15]. BC-CSCs were stocked at early passage numbers for use in further experimental step [16].

## Antiproliferative effects of 5-Fluorouracil and Doxorubucin

Doxorubucin (Dox) and 5-Fluorouracil (5-Fu) are pharmacological agents frequently used in conventional chemotherapy. Dox, which belongs to anthracycline family, is one of the most widely used drugs in breast cancer. It inhibits DNA synthesis by intercalating DNA helix. This inhibition is executed by preventing the advance of enzyme topoisomerase II along DNA molecule. During replication, having stabilized topoisomerase, DNA double-helix structure becomes unable to be resealed and the cell undergoes apoptosis [3]. 5-FU is an analogue of uracil with a fluorine atom at the C-5 position in place of hydrogen. It rapidly enters the cell using the same facilitated transport mechanism as uracil. 5-FU is converted intracellularly to several active metabolites: fluorodeoxyuridine monophosphate, fluorodeoxyuridine triphosphate and fluorouridine triphosphate. These active metabolites disrupt RNA synthesis and the action of thymidylate synthase [17].

In polychemotherapeutic approach, which is the subject of this research, more detailed information is needed about the combined effects of 5-Fu and Dox. Because it is known that combination therapy is important in the treatment of BC and significantly reduces the risk of recurrence[2]. Additionally, especially today, it is of great importance to clearly reveal the effects of these chemotherapeutic compounds and their combinations on cancer stem cells and three-dimensional tumoral structures.

In the present study conducted in this context, the antiproliferative effects of 5-Fu and Dox (alone and in combination) on the MCF-7 cell line and MCF-7 derived cancer stem cells were demonstrated on monolayer cells. Thus, experimental knowledge was established for the study to be carried out on tumor spheroids in the following steps. Within this experimental setup, in vitro antiproliferative effects of 5-Fu and Dox against BC cells and BC-CSCs (CD24^−^ and CD44^+^) were evaluated by MTT (3-(4,5-dimethylthiazol-2-yl)- 2,5-diphenyltetrazolium bromide) analysis[16]. The MTT test was carried out at different eight concentrations (Table 2). The cell lines were left in contact with two drugs and their combination for two days. IC50 concentration that inhibits cell growth by 50% was estimated by a nonlinear regression analysis using GraphPad Prism 8.0 Software. Consequently, the percentage of cell viability was calculated.

**Table 2:** 5-Fu and Dox doses used in the MTT analysis

	Dose 1	Dose 2	Dose 3	Dose 4	Dose 5	Dose 6	Dose 7	Dose 8
5-Fu	200 μM	100 μM	50 μM	25 μM	12.5 μM	6.25 μM	3.125 μM	1.5625 μM
Dox	2 μM	1 μM	0.5 μM	0.25 μM	0.125 μM	0.06 μM	0.03 μM	0.015 μM
Combination	1:1 mixture of 200 μM 5-Fu and 2 μM Dox *(highest application dose in combination)*							
	A total of eight experimental doses prepared by diluting the highest dose by 50% each time							

To evaluate the possible synergistic and antagonistic of the chemotherapeutics that used in the present study (5-FU and DOX) on breast cancer treatment, the combination index (CI) was calculated using the Chou-Talalay method with CompuSyn Software based on the results of MTT analysis at 72 h [18]. CI was plotted on the y-axis as a function of effect level (Fa) on the x-axis to assess drug synergism. The fractional effect is a value between 0 and 1, where 0 means that the drug did not affect cell viability and 1 means that the drug produced a full effect on decreasing cell viability. CI < 1 and CI > 1 (Fa = 0.5) indicated synergistic and antagonistic effects, respectively. Fractional inhibition (Fa) of 0.5 represents 50% growth inhibition in this computational data.

## Effects of 5-Fluorouracil and Doxorubucin on 3D tumor spheroids

Three-dimensional tumor spheroids were used as a model to investigate the activities of 5-Fu and Dox in this study. This model, was created with breast cancer cells (MCF-7) and their stem cells (CD24^−^/CD44^+^) to better understand the effectiveness of combined drug therapy. In this context, the effects of chemotherapeutics, which are the subject of the study, on 3D microtissue were examined by taking advantage of the colony formation activity of these cells in soft agar[19]. In this technique, homogeneous wells in terms of depth and diameter are created on the agarose gel using molds called "3D Petri Dish® " (Sigma, USA). When cells are cultured in these wells, micro-tissues are formed as a result of the process of self-assembly and adhesion over time [20]. Initially, 2% agarose solution was prepared in aqueous solution containing 0.95% NaCl. After the prepared agarose solution was sterilized for 20 min at 121 °C and 1.02 atm pressure, it was placed in 3D Petri Dish® molds in a laminar flow sterile cabinet and allowed to polymerize. The polymerized gel molds were placed in 24 well sterile culture dishes. MCF-7-derived breast cancer stem cells (CD24^−^/CD44^+^) were inoculated at a concentration of 5 × 10^5^ cells/60 µL per gel in three replicates, and cell matrices were incubated at 37°C and 5% CO_2_. Micro-tissues were photographed every 24 h and analyzed in the ImageJ program, and tissue diameters were calculated. When the diameter of the micro-tissues reached approximately 400 µm, 5-Fu, Dox and their combinations were added to them at the effective doses (IC50) determined in the previous step. The changes in the diameter of the micro-tissues were recorded by photographing them. The size analysis of the photographs taken from the culture followed for 120 h (5 days) was made in the "Image J" program, and the change in micro-tissue diameter over time was plotted.

## Effects of 5-Fluorouracil and Doxorubucin on expression of apoptotic genes

In this part of the study, qRT-PCR analysis was performed on RNAs isolated from CSC-derived 3D tumor spheroids and monolayer cells treated with 5-Fu, Dox and combined drug doses for five days in the previous step. In this context, it was investigated how CD24^−^/CD44^+^ breast cancer stem cells would respond to relevant drug applications in 3D culture conditions rather than monolayer culture. Changes in the expression of genes related to the apoptosis mechanism (Bax, Bcl-2, p53, Caspase 7,8 and 9), which has a very important place in cancer, were determined in this molecular analysis. The qRT-PCR method used in stem cell characterization and explained in the relevant section above was used in this step as well. qRT-PCR analysis was performed using cDNAs synthesized from RNAs isolated from tumor spheroids by the primer pairs specified in Table 3. Comparative ΔΔCT method was used for the quantification of amplification products obtained by real-time PCR.

**Table 3:** Primer sequences used in expression analysis of apoptotic genes

Gene Names	Forward	Reverse
*P53*	5’-GCCCAACAACACCAGCTCCT-3’	5’-CCTGGGCATCCTTGAGTTCC-3’
*Bcl-2*	5’-ATCGCCCTGTGGATGACTGAG-3’	5’-CAGCCAGGAGAAATCAAACAGAGG- 3’
*Bax*	5’-GGACGAACTGGACAGTAACATGG 3’	5’-GCAAAGTAGAAAAGGGCGACAAC-3’
*Caspase 7*	5’-CGGAACAGACAAAGATGCCGAG-3	5’-AGGCGGCATTTGTATGGTCCTC-3’
*Caspase-8*	5’-AGAAGAGGGTCATCCTGGGAGA-3	5’-TCAGGACTTCCTTCAAGGCTGC-3’
*Caspase-9*	5’-GTTTGAGGACCTTCGACCAGCT-3’	5’-CAACGTACCAGGAGCCACTCTT-3’
*GAPDH (housekeeping gene)*	5’-GGATTTGGTCGTATTGGG-3’	5’-GGAAGATGGTGATGGGATT-3’

## Statistical analysis

All experiments were repeated three times. All values were expressed using GraphPad Prism 9 Statistical Software. Experimental results were analysed by Two-Way ANOVA. Additionally, Bonferroni-Sidak's post hoc analysis is performed by the same software. value of < 0.05 was considered statistically significant.

## Results

### Breast *cancer* stem cell ısolation and characterization

**Fig. 1:**
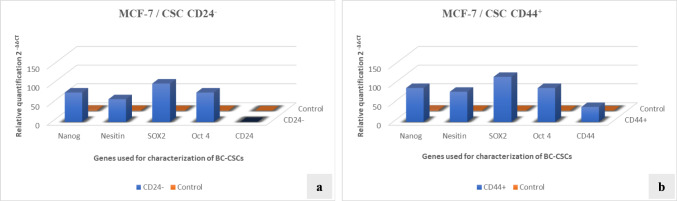
Characterization of breast cancer stem cells derived from MCF-7 cell lines. *[Gene expression levels in CD24*^*−*^* CSCs (a) and CD44*.^+^ *CSCs (b)]*

Breast cancer stem cells (BC-CSCs) isolated from MCF-7 cell line by MACS magnetic cell separation device were characterized by qRT-PCR. In this experimental step, the expression levels of stemness related transcription factors known as *SOX2, OCT4, Nanog* and *Nestin* were quantified. In addition, the expression levels of surface markers used during cell isolation were also analyzed, and thus, the reliability of the CSC separation method was confirmed. The data obtained have been graphed in Fig. 1.

In the presented graphs, it was observed that the gene regions of stemness factors and surface markers used in the characterization of cancer stem cells isolated from the MCF-7 breast cancer cell line were significantly expressed. The lack of expression of the relevant surface marker on CD24^−^ stem cells demonstrated the accuracy and reliability of the isolation method. As a result, it has been proven at the molecular level that these cells are cancer stem cells.

## Antiproliferative effects of 5-Fluorouracil and Doxorubucin

**Fig. 2:**
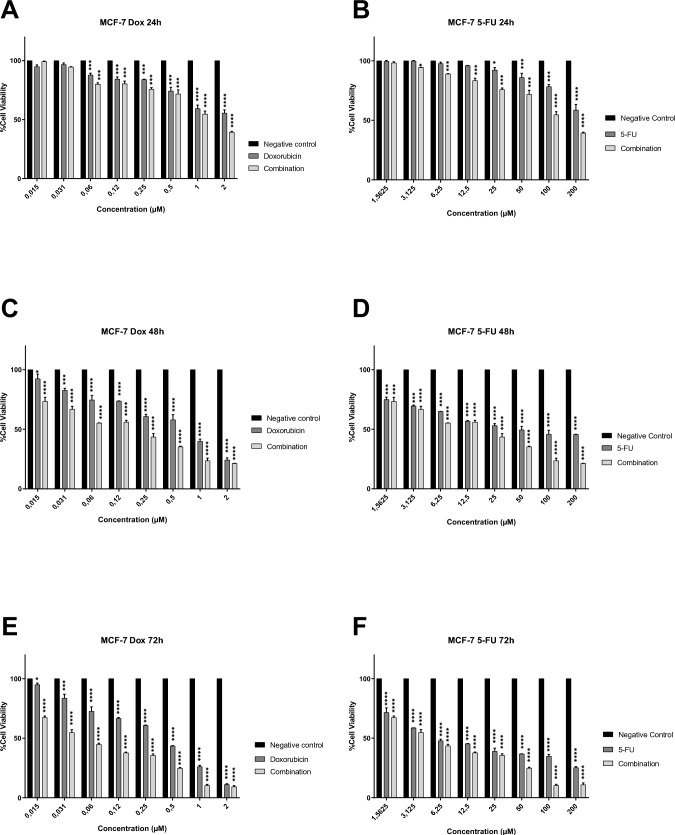
The antiproliferative effects of Dox (A, C and E) and 5-Fu (B, D and F) on the MCF-7 cell line *(The combined effect of both drugs is shown in the graphs a a third column for each experimental dose).* Significant differences were obtained at *P < 0.05 and ****P < 0.0001 using two-way ANOVA

**Fig. 3:**
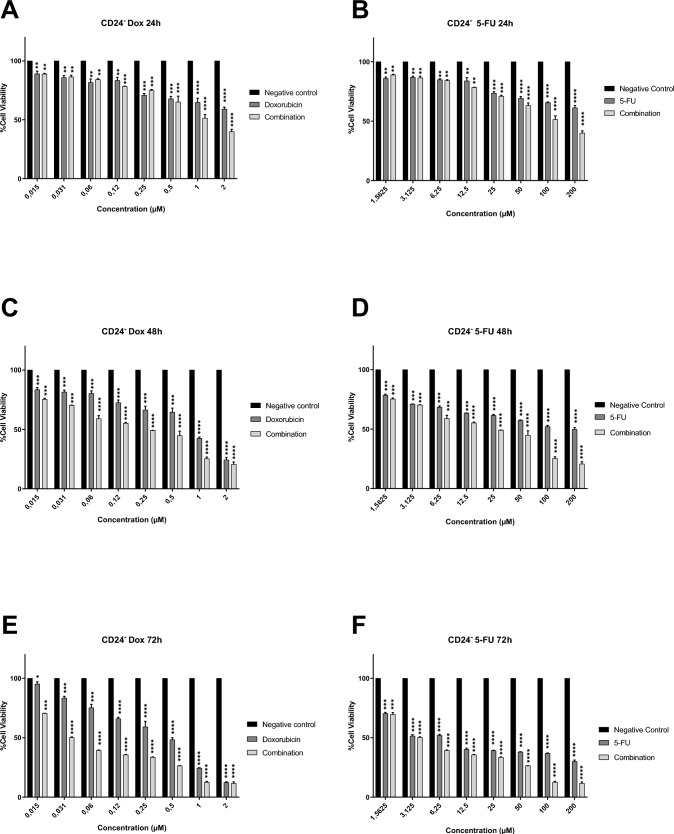
The antiproliferative effects of Dox (A, C and E) and 5-Fu (B, D and F) on the CD24^−^ breast cancer CSCs *(The combined effect of both drugs is shown in the graphs a a third column for each experimental dose).* Significant differences were obtained at *P < 0.05, **P < 0.01, ***P < 0.001 and ****P < 0.0001 using two-way ANOVA

**Fig. 4:**
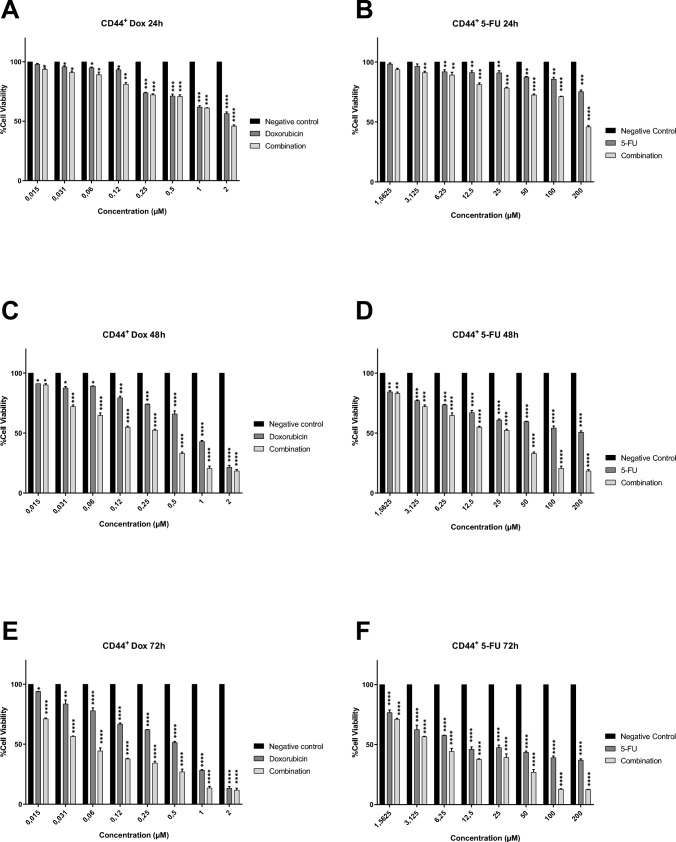
The antiproliferative effects of Dox (A, C and E) and 5-Fu (B, D and F) on the CD44^+^ breast cancer CSCs *(The combined effect of both drugs is shown in the graphs a a third column for each experimental dose).* Significant differences were obtained at *P < 0.05, **P < 0.01, ***P < 0.001 and ****P < 0.0001 using two-way ANOVA

In the present study, the antiproliferative effects of 5-Fu and Dox (alone and in combination) on the MCF-7 cell line (Fig. 2) and MCF-7 derived CSCs (Fig. 3 and 4) were demonstrated on monolayer cells.

As seen in Fig. 2, a significant antiproliferative effect was observed in all experimental groups (5-Fu, Dox and combination) in the monolayer culture of the MCF-7 cell line. At the end of the 72nd hour, the cell viability percentages determined reached significantly low levels. When all incubation periods were evaluated, it was determined that the most effective dose for 5-Fu was 200 µM, 2 µM for Dox and Dose 1 (Table 2, 1:1 mixture of 200 μM 5-Fu and 2 μM Dox) for the drug combination. In addition, according to the data obtained at 48 and 72 h, it was determined that all application doses inhibited the proliferation and viability of breast cancer cells in a statistically significant manner. When the trial groups were evaluated within themselves, it was determined that doxorubicin was more effective than 5-fluorouracil on the MCF-7 cell line, and the combined application showed the highest antiproliferative effect. This clearly demonstrates that multiple chemotherapeutic applications provide more effective results on breast cancer than monotherapies.

When the graphs in Figs. 3 and 4 were examined, it was seen that breast cancer stem cells (CD24^−^ and CD44^+^) cultured in two-dimensional monolayers also respond to all application doses. However, unlike MCF-7, they were found to be more resistant, and therefore, their % cell viability was higher. When the above mentioned doses (200 µM for 5-Fu, 2 µM for Dox and Dose 1 for combination) were evaluated for all incubation periods, it was observed that the viability levels were approximately 40–70% at the end of the 24th hour, 25–60% at the end of the 48th hour, and decreased to 20% at the end of 72 h. In addition, it was determined that all application doses inhibited the proliferation and viability of breast cancer stem cells in a statistically significant manner. When the trial groups were evaluated within themselves, it was determined that doxorubicin was more effective than 5-fluorouracil on breast cancer stem cells, and the combined application showed the highest antiproliferative effect. This clearly demonstrates that multiple chemotherapeutic applications provide more effective results on breast cancer cancer stem cells.

In line with the data presented above, IC50 concentration that inhibits cell growth by 50% was estimated by a nonlinear regression analysis using GraphPad Prism 8.0 Software (Table 4). It was determined that the IC50 values calculated for cancer stem cells were higher than those for MCF-7. In determining the IC50s of combined drugs, the IC50s of Dox and 5-Fu in the combination were calculated separately. Accordingly, a much lower IC50 was reached compared to the IC50 calculated when used alone. In the next analysis steps, an experimental setup was made with these values. For example, 5.42 µM 5-Fu, 7.34 µM Dox and 1:1 mixture of Dox and 5-Fu (3.27 and 4.98, respectively) were applied to 3D tumor spheroids created with CD24^−^ CSCs.

**Table 4:** IC50 doses for all experimental groups in breast cancer cells and their CSCs

	5-fluorouracil (µM)	Doxorubicin (µM)	Combination (µM)
*(Dox and 5-Fu, respectively)*			
MCF-7 cell line	0.25	0.8	0.17 and 0.65
MCF-7 derived CD24^−^ CSCs	5.42	7.34	3.27 and 4.98
MCF-7 derived CD44^+^ CSCs	6.57	8.16	4.05 and 5.84

**Fig. 5:**
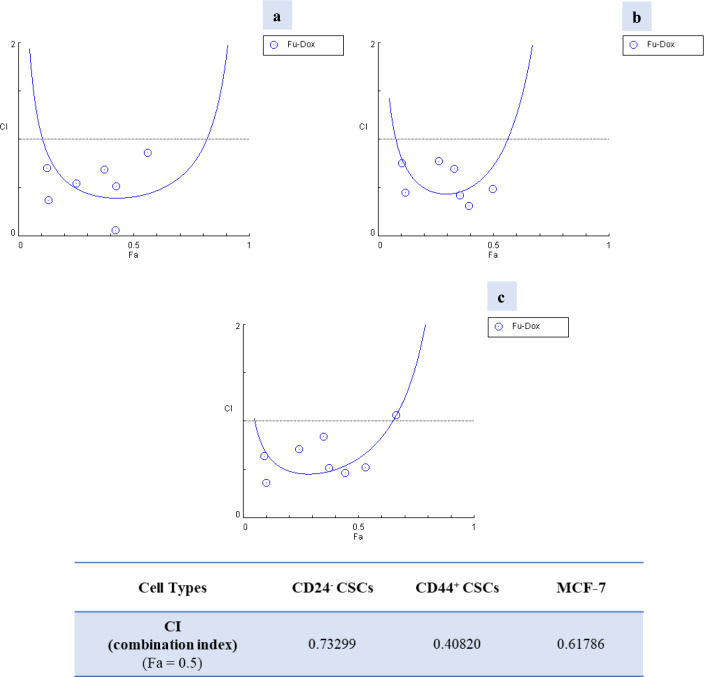
The combination effects of 5-FU and Dox on CD44^+^CSCs (a), CD24^−^CSCs (b) and MCF-7 cells (c) in vitro

The combination index (CI) values were determined by the Chou-Talalay equation with CompuSyn Software. Since the CI values calculated in all cell groups used in the study (MCF-7, CD24^−^CSCs and CD44^+^CSCs) were below 1 (Fig. 5, Fa = 0.5), it was clearly demonstrated that 5-Fu and Dox had a synergistic effect on breast cancer.

## Effects of 5-Fluorouracil and Doxorubucin on 3D tumor spheroids

**Fig. 6:**
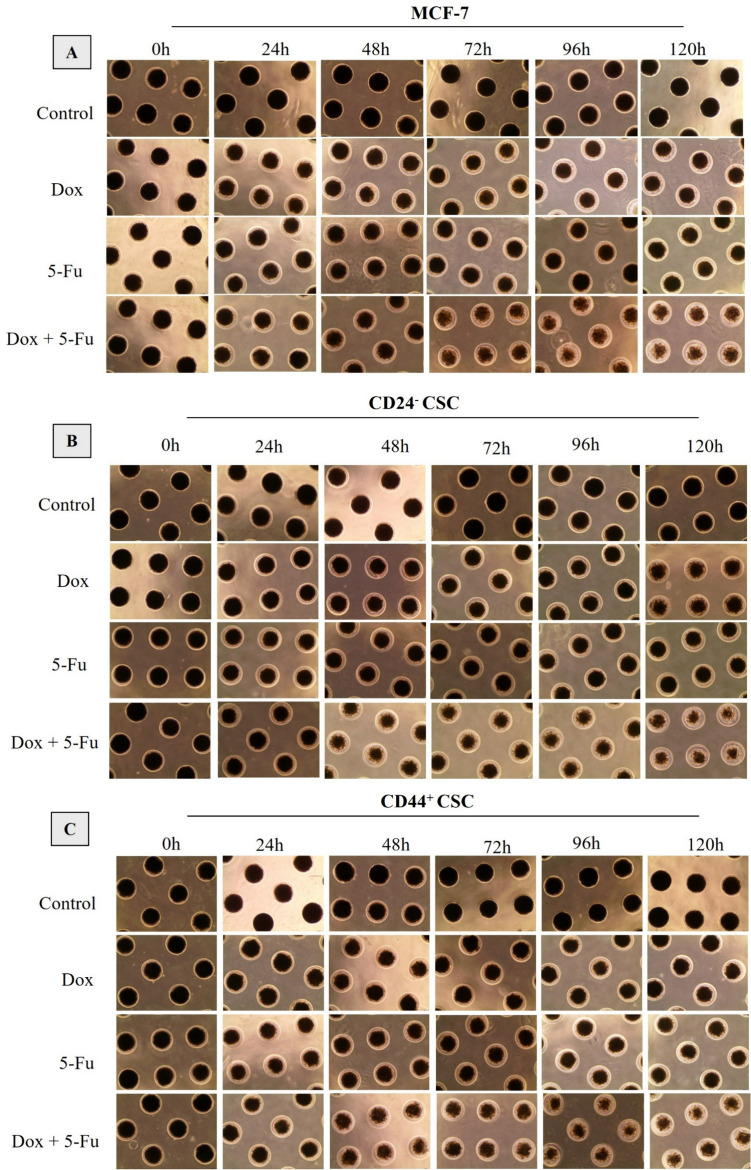
Effects of 5-Fluorouracil and Doxorubucin on 3D tumor spheroids. (A- MCF-7 spheroids, B-CD24^−^ CSC spheroids and C- CD44.^+^ CSC spheroids, 4 × magnification)

3D tumor spheroids were created with MCF-7 and BC-CSCs. In this context, effective IC50 doses determined in the monolayer antiproliferative analysis were applied to microspheres and the cells were treated with drugs in their three-dimensional microenvironment for 120 h. Microscope images taken every 24 h during this period have been presented in Fig. 6.

When Fig. 6 was examined, it was seen that Dox was more effective than 5-Fu application on both MCF-7 and CSC (CD24^−^ and CD44^+^) spheroids and caused a more significant shrinkage in 3D tumor models. According to the photographs taken from the microscope, it was observed that the most effective result was obtained in combined drug application. When the change in the diameters of the 3D tumor spheroids was examined temporally, it was seen that the shrinkage started from the 24th hour in all groups, and the most effective drug application here was the Dox/5-Fu composition. According to the photographs in Fig. 6, similar shrinkage rates were observed in MCF-7, CD24^−^CSCs and CD44^+^CSCs derived tumoroids. This result did not mean that there was no difference in chemotherapeutic resistance between these groups. On the contrary, when the IC50 values stated in Table 4 have been examined, it has been seen that the IC50 doses of each group are different. Therefore, it should not be forgotten that different chemotherapeutic doses are applied to spheroids formed with different cell groups. On the contrary, when the effective inhibition doses specified in Table 4 are examined, the IC50 doses of each group are different; therefore, it should not be ignored that different chemotherapeutic doses are applied to spheroids formed with different cell groups. It can be clearly seen that a difference similar to the shrinkage observed in MCF-7 tumoroids occurs only with much higher doses in the CSC groups. In this examination, which was carried out with IC50 values obtained by MTT analysis in 2D monolayer culture, a much longer period of time (120 h) was needed for significant tumoroid shrinkage. This shows that antiproliferative analyzes performed in 3D tumor models reveal much different perspectives compared to 2D.

**Fig. 7:**
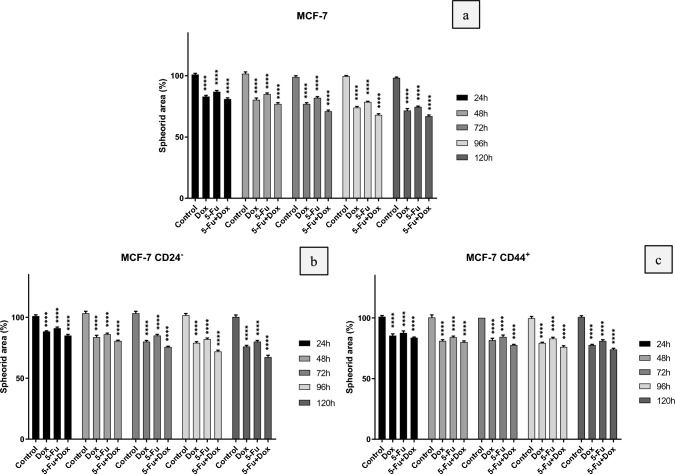
The changes in the diameter of the micro-tissues after drug administration. (a-MCF-7 spheroids, b-CD24^−^ CSC spheroids and c- CD44^+^ CSC spheroids, Significant differences were obtained at ***P < 0.001 using two-way ANOVA)

The size analysis of the photographs was made in the "Image J" program, the changes in the diameter of the micro-tissues were calculated and plotted (Fig. 7). When the created graphics were examined, it was clearly seen that the evaluations made regarding the images in Fig. 6 were confirmed. It was statistically determined that the amount of tumor shrinkage in all experimental groups was significantly different from the control groups.

In the other part study, qRT-PCR analysis was performed on RNAs isolated from CSC-derived 3D tumor spheroids and monolayer cells treated with 5-Fu, Dox and combined IC50 doses for five days in the previous steps. Changes in the expression of genes related to the apoptosis mechanism (Bax, Bcl-2, p53, Caspase 7,8 and 9) were calculated relative to control group, and then, graphs were created with the determined 2^−ΔΔCt^ values.

**Fig. 8:**
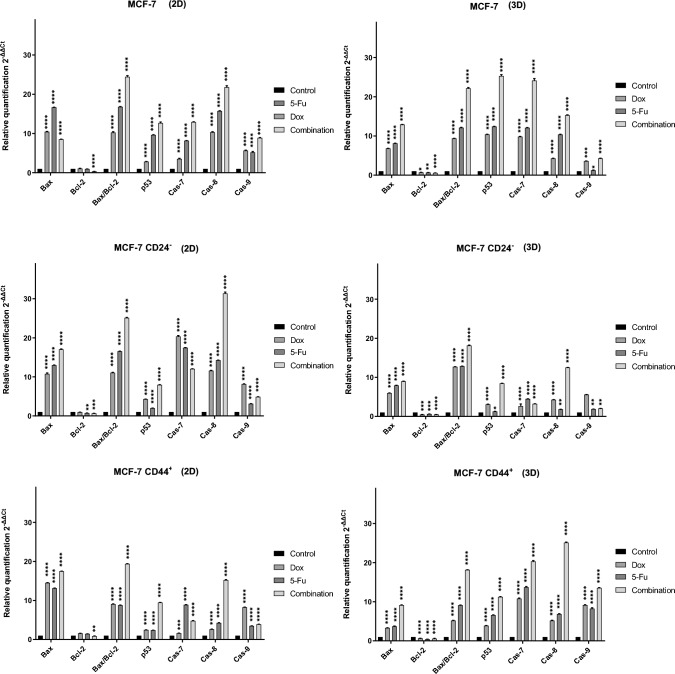
Effects of 5-FU and Dox on the expression of apoptotic genes in 2D and 3D culture conditions. (Significant differences were obtained at *P < 0.05, **P < 0.01, ***P < 0.001 and ****P < 0.0001 using two-way ANOVA.)

When the graphs presented in Fig. 8 were examined, it was seen that all chemotherapeutic prescriptions significantly increased the apoptosis-related gene expression levels in tumor spheroids. Especially, the increase in Bax, Bax/Bcl-2, and caspase groups (7–8 and 9) was clearly revealed. It has been observed that combined drug administration generally changes the expression of relevant genes more clearly than 5-Fu or Dox alone. When the data obtained from tumoroids created with breast cancer stem cells and the MCF-7 cell line were compared, it was determined that BC-CSCs were more resistant in this analysis and the increase in gene expression was relatively less. Here, as in the previous step, it should not be ignored that the IC50 doses of each group are different (Table 4) and that higher doses of drugs were applied to the cancer stem cells. Additionally, when all experimental groups were compared within themselves in terms of culture systems (2D and 3D), it was molecularly demonstrated that 3D tumoroids were more resistant to apoptosis.

## Discussion

In the present study, the MACS magnetic cell separation device system used in breast cancer stem cell isolation worked successfully in accordance with the literature [12, 13, 21]. In characterization studies, the obtained results were found to be compatible with the studies reported in previous years[14].

In antiproliferative activity studies carried out under monolayer (2D) culture conditions, significant cell death in cancer cells was determined for all drug applications. In this context, in line with the literature, it has been determined that Dox is more effective than 5-Fu[3]. It inhibited cell viability and proliferation even at low doses. Experimental data obtained in this study showed that doxorubicin was more effective than 5-fluorouracil on both the MCF-7 cell line and breast cancer stem cells. When the literature was scanned to explain this result, it was seen that ER-positive/HER2-negative tumors had high chemosensitivity to DOX [22, 23]. The relevant data are consistent with the result obtained in this study using MCF-7, an ER-positive/HER2-negative breast cancer cell line. Therefore, in this study, it is thought that one of the reasons why the antiproliferative activity of DOX is higher than 5-Fu may be the characteristic feature of the MCF-7 cell line (ER-positive/HER2-negative). Additionally, the combined drug preparation has a higher antiproliferative effect than the single drug[2]. Similar results were obtained in 3D culture conditions; similar results were obtained with 2D culture by longer exposure time (120 h) and higher doses of drugs. This result is consistent with the scientific article published by Gong et al. in 2015[9]. In addition, when 2D and 3D culture conditions were evaluated together, it was determined that breast cancer stem cells were more resistant than the MCF-7 cancer cell line and required higher drug doses and longer incubation periods to observe a significant antiproliferative effect [24, 25]. This result also shows high compatibility with the relevant literature [4, 6, 23, 24]. In this study designed with MCF-7, a HER2-negative breast cancer cell line, the observation of a synergistic effect for 5-Fu and Dox is of extra importance, together with the data presented in another study published by Mody et al. in 2023. According to this publication, when DOX was administered with another drug that has a synergistic effect with it, side effect markers decreased in the HER2-positive breast cancer cell line, while such an effect was not observed in the MCF-7 cell line [27]. This shows how important breast cancer types are for treatment, and therefore the in vitro results of synergistic or antagonistic effects may produce misleading results when transferred to the clinic. Therefore, the data in this manuscript will make a significant contribution to the studies on the transfer of chemotherapeutics, which have synergistic effects, specifically Dox and 5-Fu, to the clinic [28].

The possible effects of Dox and 5-Fu compounds (as monotherapy and in combination) on the relative expression of genes related to apoptosis were studied. In this context, the expression levels of Bax, Bcl-2 and some caspases were analyzed. As a result, data showed that these drugs (Dox and 5-Fu), alone or together, increased the expression of Bax and Bax/Bcl-2, revealing that the apoptosis mechanism was induced. The data obtained are very valuable, especially since clinically increasing Bax levels are associated with a good response to chemotherapy [29]. Therefore, the experimental process carried out in this study ended with results consistent with the literature [26, 27]. The expressional activities of caspases, another enzyme family involved in apoptosis, known as one of the important mechanisms used in cancer treatment, against the applied chemotherapeutic drug doses were also examined in this study. Changes in the expression levels of caspase 7, 8 and 9, which have an important place in this process, were specifically examined and it was determined that all of them were upregulated, consistent with the literature [28–30]. Caspases-8 and 9 are known as initiators of the apoptosis process. However, activation of caspase-8 constitutes a crucial step in the initiation of the death receptor-mediated apoptosis pathway (extrinsic pathway) [31–33]. Caspase-9 affects the apoptotic process by playing a very important role in the intrinsic pathway mediated by mitochondria[34]. Considering this basic molecular information, in this study, it has been clearly seen that apoptosis was activated through both extrinsic and intrinsic pathways.

In addition, it has been seen that apoptosis-resistant phenotype associated with 3D tumor spheroids is caused primarily by growth, drug penetrance, or lower oxygen tension and induction of hypoxia in cells embedded within multicellular tumors. For example, hypoxia and reduced cell growth could lead to loss of functional p53 and impede stress-induced apoptosis induction. Because cells incorporated into 3D tissue structures are able to effectively resist apoptosis, and life and death decisions depend upon a delicate balance between proapoptotic and anti-apoptotic signals, cell adhesion in 3D must titrate cell fate in favor of cell survival. Mitochondrial integrity is central to apoptosis regulation, and therefore, it is highly likely that tissue architecture enhances cell survival by modulating mitochondria homeostasis [35–37].

In conclusion, the polychemotherapeutic approach was much more effective than the monotherapy. The fact that this effect was seen not only in breast cancer cells, but also in breast cancer stem cells. In addition, it was very promising that the results obtained were similar in both two-dimensional and three-dimensional tumoroids.

## Data Availability

The data generated in the present study may be requested from the corresponding author.

## References

[1] Comşa Ş, Am C, Raıca M (2015). The story of MCF-7 breast cancer cell line: 40 years of experience in research. Anticancer Res.

[2] Fisusi FA, Akala EO (2019). Drug combinations in breast cancer therapy. Pharm Nanotechnol.

[3] Eroglu O, Kaya H, Celik EG (2019). Triple effect of doxorubicin, 5-fluorouracil, propranolol on cell survival on MCF-7 breast cancer cell line. J Biosci Med.

[4] Byus G (2023). Breast cancer stem cells & cancer stem cells and their treatments. Microreviews Cell Mol Biol.

[5] Muduli K, Prusty M, Pradhan J (2023). Estrogen-related receptor alpha (ERRα) promotes cancer stem cell-like characteristics in breast cancer. Stem Cell Rev Reports.

[6] Wu M, Zhang X, Zhang W (2023). Paracrine secretion of IL8 by breast cancer stem cells promotes therapeutic resistance and metastasis of the bulk tumor cells. Cell Commun Signal.

[7] Ozturk S, Gorgun C, Gokalp S (2020). Development and characterization of cancer stem cell-based tumoroids as an osteosarcoma model. Biotechnol Bioeng.

[8] Fröhlich E (2023). The variety of 3D breast cancer models for the study of tumor physiology and drug screening. Int J Mol Sci.

[9] Gong X, Lin C, Cheng J (2015). Generation of multicellular tumor spheroids with microwell-based agarose scaffolds for drug testing. PLoS ONE.

[10] Okuyama NCM, Ribeiro DL, da Rocha CQ (2023). Three-dimensional cell cultures as preclinical models to assess the biological activity of phytochemicals in breast cancer. Toxicol Appl Pharmacol.

[11] Andrahennadi S, Sami A, Manna M (2021). Current landscape of targeted therapy in hormone receptor-positive and HER2-negative breast cancer. Curr Oncol.

[12] Wang J, Cao MG, You CZ (2012). A preliminary investigation of the relationship between circulating tumor cells and cancer stem cells in patients with breast cancer. Cell Mol Biol.

[13] Jariyal H, Gupta C, Bhat VS (2019). Advancements in cancer stem cell isolation and characterization. Stem cell Rev reports.

[14] Neradil J, Veselska R (2015). Nestin as a marker of cancer stem cells. Cancer Sci.

[15] Bilgin S, Tayhan SE, Yıldırım A, Koc E (2023). Investigation of the effects of isoeugenol-based phenolic compounds on migration and proliferation of HT29 colon cancer cells at cellular and molecular level. Bioorg Chem.

[16] Erden Tayhan S, Bilgin S, Yıldırım A, Koç E (2023). Biological screening of polyphenol derivatives for Anti-Proliferative, Anti-Apoptotic and Anti-Migrative activities in human breast cancer cell lines MCF-7. Chem Biodivers.

[17] Longley DB, Harkin DP, Johnston PG (2003). 5-Fluorouracil: mechanisms of action and clinical strategies. Nat Rev Cancer.

[18] Gökşen Tosun N (2023). Enhancing therapeutic efficacy in breast cancer: a study on the combined cytotoxic effects of doxorubicin and MPC-3100. Naunyn Schmiedebergs Arch Pharmacol.

[19] Bielecka ZF, Maliszewska-Olejniczak K, Safir IJ (2017). Three-dimensional cell culture model utilization in cancer stem cell research. Biol Rev.

[20] Achilli T-M, Meyer J, Morgan JR (2012). Advances in the formation, use and understanding of multi-cellular spheroids. Expert Opin Biol Ther.

[21] Dobbin ZC, Landen CN (2013). Isolation and characterization of potential cancer stem cells from solid human tumors—potential applications. Curr Protoc Pharmacol.

[22] Ahn SG, Bae SJ, Yoon C (2017). Chemosensitivity to doxorubicin of ER-positive/HER2-negative breast cancers with high 21-gene recurrence score: a study based on in vitro chemoresponse assay. PLoS ONE.

[23] Yao L, Liu Y, Li Z (2011). HER2 and response to anthracycline-based neoadjuvant chemotherapy in breast cancer. Ann Oncol.

[24] Mazurakova A, Koklesova L, Vybohova D (2023). Therapy-resistant breast cancer in focus: clinically relevant mitigation by flavonoids targeting cancer stem cells. Front Pharmacol.

[25] Zheng Q, Zhang M, Zhou F (2021). The breast cancer stem cells traits and drug resistance. Front Pharmacol.

[26] Cho Y, Kim YK (2020). Cancer stem cells as a potential target to overcome multidrug resistance. Front Oncol.

[27] Mody H, Vaidya TR, Lezeau J (2023). In vitro to clinical translation of combinatorial effects of doxorubicin and dexrazoxane in breast cancer: a mechanism-based pharmacokinetic/pharmacodynamic modeling approach. Front Pharmacol.

[28] Frankman ZD, Jiang L, Schroeder JA, Zohar Y (2022). Application of microfluidic systems for breast cancer research. Micromachines.

[29] Contadini C, Ferri A, Cirotti C (2023). Caspase-8 and Tyrosine Kinases: a dangerous liaison in cancer. Cancers (Basel).

[30] Salavatipour MS, Kouhbananinejad SM, Lashkari M (2023). Kermanian propolis induces apoptosis through upregulation of Bax/Bcl-2 ratio in acute myeloblastic leukemia cell line (NB4). J Cancer Res Ther.

[31] Önder GÖ, Göktepe Ö, Baran M (2023). Therapeutic potential of hesperidin: apoptosis induction in breast cancer cell lines. Food Chem Toxicol.

[32] Mohany KM, Abdel Shakour AB, Mohamed SI (2023). Cytotoxic n-hexane fraction of the egyptian pteris vittata functions as anti-breast cancer through coordinated actions on apoptotic and autophagic pathways. Appl Biochem Biotechnol.

[33] Teng J-W, Hung E, Wu J-M (2023). Anti-tumor effects of IL-1β induced TRAIL-expressing hUCMSCs on embelin treated breast cancer cell lines. Asian Pacific J Cancer Prev.

[34] Kaproń B, Czarnomysy R, Radomska D (2023). Thiosemicarbazide derivatives targeting human TopoIIα and IDO-1 as Small-molecule drug candidates for breast cancer treatment. Int J Mol Sci.

[35] Girard YK, Wang C, Ravi S (2013). A 3D fibrous scaffold inducing tumoroids: a platform for anticancer drug development. PLoS ONE.

[36] Zahir N, Weaver VM (2004). Death in the third dimension: apoptosis regulation and tissue architecture. Curr Opin Genet Dev.

[37] Kim JW, Ho WJ, Wu BM (2011). The role of the 3D environment in hypoxia-induced drug and apoptosis resistance. Anticancer Res.

